# Deciphering of Genomic Loci Associated with Alkaline Tolerance in Soybean [*Glycine max* (L.) Merr.] by Genome-Wide Association Study

**DOI:** 10.3390/plants14030357

**Published:** 2025-01-24

**Authors:** Xinjing Yang, Ye Zhang, Javaid Akhter Bhat, Mingjing Wang, Huanbin Zheng, Moran Bu, Beifang Zhao, Suxin Yang, Xianzhong Feng

**Affiliations:** 1 Key Laboratory of Soybean Molecular Design Breeding, National Key Laboratory of Black Soils Conservation and Utilization, Northeast Institute of Geography and Agroecology, Chinese Academy of Sciences, Changchun 130102, China; yangxinjing@iga.ac.cn (X.Y.); zhangye@iga.ac.cn (Y.Z.); zhenghuanbin2024@163.com (H.Z.); bumoran@iga.ac.cn (M.B.); zhaobeifang@iga.ac.cn (B.Z.); 2 College of Advanced Agricultural Sciences, University of Chinese Academy of Sciences, Beijing 100049, China; 3 Zhejiang Lab, Hangzhou 310012, China; javid.akhter69@gmail.com (J.A.B.); wangmingjing2021@163.com (M.W.)

**Keywords:** soybean, alkaline tolerance, GWAS, haplotype, candidate gene

## Abstract

Alkaline stress is one of the major abiotic constraints that limits plant growth and development. However, the genetic basis underlying alkaline tolerance in soybean [*Glycine max* (L.) Merr.] remains largely unexplored. In this study, an integrated genomic analysis approach was employed to elucidate the genetic architecture of alkaline tolerance in a diverse panel of 326 soybean cultivars. Through association mapping, we detected 28 single nucleotide polymorphisms (SNPs) significantly associated with alkaline tolerance. By examining the genomic distances around these significant SNPs, five genomic regions were characterized as stable quantitative trait loci (QTLs), which were designated as *qAT1*, *qAT4*, *qAT14*, *qAT18,* and *qAT20*. These QTLs are reported here for the first time in soybean. Seventeen putative candidate genes were identified within the physical intervals of these QTLs. Haplotype analysis indicated that four of these candidate genes exhibited significant allele variation associated with alkaline tolerance-related traits, and the haplotype alleles for these four genes varied in number from two to four. The findings of this study may have important implications for soybean breeding programs aimed at enhancing alkaline tolerance.

## 1. Introduction

Climate change has become a substantial threat to agriculture production by increasing the occurrence of both biotic and abiotic stress events [1]. Saline–alkaline stress has impacted about 20% of cultivated land in the world, which is extensively dispersed across more than 100 countries and covers 800 million hectares [2]. Soybean [*Glycine max* (L.) Merr.], the fifth most important crop after wheat, maize, rice, and potato, is extensively cultivated and consumed worldwide [3]. Among environmental challenges, alkaline stress has been recognized as an important detrimental factor for soybean productivity [4,5]. Studies have documented the significant correlations of alkaline stress with shoot and root biomass-related traits in soybean [6]. However, there is a lack of detailed information of the genetic basis that regulates the alkaline tolerance in soybean. Therefore, a comprehensive understanding of the genetic factors controlling the alkaline tolerance in soybean will allow their deployment in the production of alkaline-tolerant soybean cultivars [7,8].

Previously, the efforts put forward on unraveling the genetic factors governing the alkaline tolerance in soybean were minimal, and only four QTLs associated with alkaline stress tolerance have been documented in SoyBase (https://www.soybase.org/). The genome-wide association study (GWAS) approach, possessing a high resolution and precision, has emerged as the practical method in marker-assisted breeding applications [9,10]. In soybean, GWAS has been performed for the deciphering of genomic loci associated with different traits. This approach has been employed to investigate various forms of tolerance in soybean, including salt tolerance in diverse soybean accessions [11,12], drought tolerance among a panel of 259 Chinese soybean cultivars [13], seed-flooding tolerance in 347 diverse soybean accessions [14], and cold tolerance during the germination stage in 260 soybean accessions [15]. However, there has been relatively limited application of GWAS to examine alkaline stress tolerance in soybean [9,16].

To investigate alkaline tolerance at the genetic level, this study utilized an association mapping panel consisting of 326 diverse soybean accessions for genetic mapping, haplotype analysis, and candidate gene identification. Major QTLs, haplotypes, and candidate genes associated with soybean alkaline tolerance were identified through integrated genomic analysis. These genetic factors could be incorporated into soybean breeding programs designed to enhance alkaline tolerance.

## 2. Results

### 2.1. Alkaline Treatment of Soybean Germplasm

**Figure 1:**
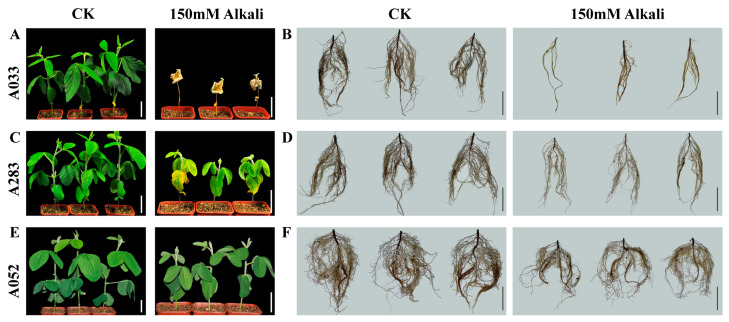
The morphological characteristics of the shoot and root of the three representative cultivars from the panel of 326 soybean cultivars under control (CK) and alkaline treatment (AT). (**A**,**B**) A033, alkaline-sensitive cultivar; (**C**,**D**) A283, moderately alkaline-tolerant cultivar; (**E**,**F**) A052, alkaline-tolerant cultivar. Scale bars: 5 cm.

According to our pre-experiment and previous reports [4,5,6], a concentration of 150 mM was used for alkaline treatment (AT) to evaluate the phenotypic performance of 326 soybean accessions, and compared to the control using Murashige and Skoog (MS) medium (CK). Two-week-old seedlings were subjected to CK and AT for one week for phenotypic observation. After seven days of treatment, the soybean germplasm accessions exhibited a range of responses to AT, from sensitive to moderately tolerant and highly tolerant. Extremely sensitive accessions experienced the wilting of aboveground leaves, plant death, and significant reductions in both shoot and root biomass compared to CK (Figure 1A,B). Accessions with moderate alkaline tolerance displayed yellowing and the wilting of leaves, as well as reductions in shoot and root biomass under AT (Figure 1C,D). However, compared to sensitive cultivars, those with moderate alkaline tolerance exhibited a lesser reduction in both shoot and root biomass when exposed to AT, while alkaline-tolerant soybean accessions showed the least impact on root and shoot biomass under AT relative to CK (Figure 1E,F).

### 2.2. Phenotypic Analysis of Alkaline Tolerance-Related Traits

Alkaline stress significantly impacts the growth and development of soybean seedlings. Eight traits were selected for evaluating alkaline tolerance at the seedling stage: seedling fresh weight (SFW), seedling dry weight (SDW), root dry weight (RDW), root fresh weight (RFW), relative chlorophyll content (CC), root number (RN), root tip number (RTN), and root length (RL). These traits exhibited substantial phenotypic variation among the 326 soybean accessions in the GWAS panel, categorized into three groups: CK, AT, and AT/CK (the ratio of the trait value under AT in compared to CK) (Table 1). Furthermore, these eight traits related to alkaline stress tolerance demonstrated highly significant correlations with one another across all three groups (Figure S1).

### 2.3. Population Structure and LD Analysis

Whole-genome resequencing analysis of the 326 soybean accessions identified 3,311,166 high-quality SNPs distributed across all 20 chromosomes of soybean for future GWAS analysis (Table S1). The lowest number of SNPs was found on chromosome 11 (62,554), while the highest was on chromosome 15 (254,495) (Table S1). Marker density varied considerably across the different chromosomes, with the highest density observed on chromosome 15 (4916 SNPs/Mb) and the lowest on chromosome 11 (1799 SNPs/Mb) (Table S1).

**Figure 2:**
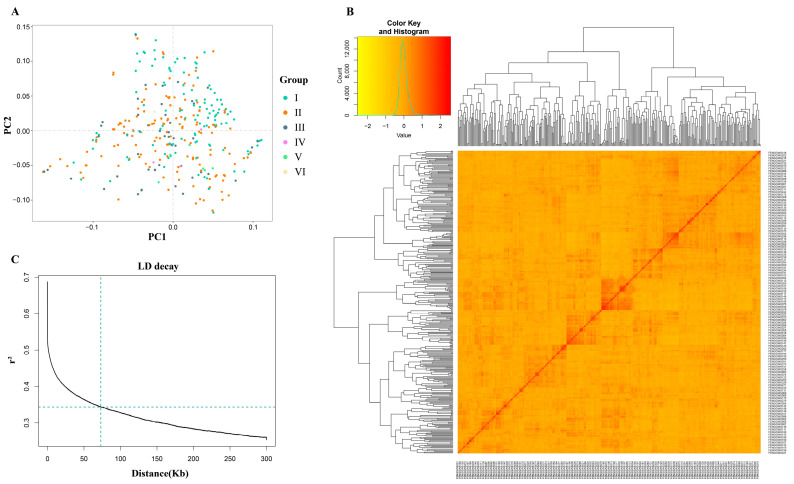
The population structure and linkage disequilibrium (LD) analysis of 326 soybean accessions. (**A**) Population structure analysis of soybean accessions; (**B**) kinship plot illustrating the relationships among soybean accessions. (**C**) LD decay plot for soybean cultivars using 3,311,166 SNP markers. The LD decay fitted with a smoothing spline regression model is represented by the curve. The intersection of the green vertical line with the horizontal green line indicates the half-decay of LD (*r*^2^ = 0.34), with the corresponding genetic distance at this point reflecting an LD decay distance of 71.6 kb.

The analysis of population structure revealed no distinct patterns, exhibiting a continuous distribution (Figure 2A; Table S2). Additionally, the kinship matrix, represented by the dendrogram and heatmap, did not demonstrate clear clustering among the soybean accessions (Figure 2B). The results of the linkage disequilibrium (LD) analysis are depicted in the graph (Figure 2C). The mean value of *r^2^* in the soybean genome was 0.68, indicating that LD began to decay at this point, with half-decay reached at *r*^2^ = 0.34. The LD decay curve intersected the half-decay point at a distance of 71.6 kb, which determines the linkage across the entire soybean genome. Thus, the genomic intervals of ±71.6 kb upstream and downstream of the stable significant SNPs are considered QTLs.

### 2.4. Association Mapping Analysis of Alkaline Tolerance-Related Traits

**Figure 3:**
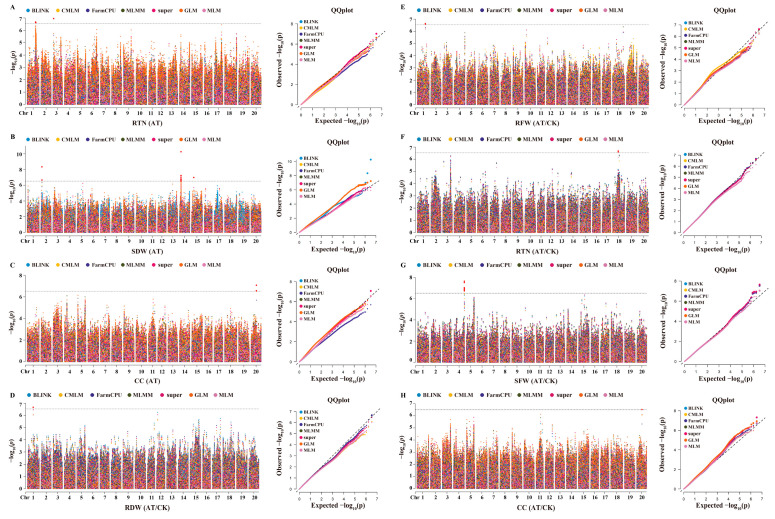
The GWAS analysis of related traits in the diverse panel of 326 soybean accessions under AT and AT/CK groups. (**A**–**C**) Manhattan and quantile–quantile (QQ) plot for RTN, SDW, and CC in the AT group, respectively. (**D**–**H**) The Manhattan and QQ plot for RDW, RFW, RTN, and CC in the AT/CK group, respectively. The horizontal dotted black line indicates the threshold level of significance (−log_10_*p* > 6.67), with soybean chromosomes represented on the X-axis.

Through GWAS analysis utilizing seven models, we identified a total of 35 SNPs significantly associated with eight traits across the three groups: CK, AT, and AT/CK (Figure 3; Table S3). In the CK group, seven significant SNPs were associated with five traits: root fresh weight (RFW) (1), root length (RL) (1), root number (RN) (1), seedling fresh weight (SFW) (1), and chlorophyll content (CC) (4). In the AT group, we detected 20 significant SNPs associated with two traits: root tip number (RTN) (3) and seedling dry weight (SDW) (17). Notably, 17 significant SNPs associated with SDW were detected exclusively in the AT group; 15 of these are located on chromosome 14 within a 100 kb region, suggesting close linkage among them. In the AT/CK group, seven significant SNPs were identified, associated with four traits: root dry weight (RDW) (1), root fresh weight (RFW) (1), root tip number (RTN) (1), and seedling fresh weight (SFW) (4). Only one significant SNP, Chr20_25660093, was associated with the CC trait in both the AT and AT/CK groups. The SNPs identified in the AT and AT/CK groups were more likely associated with alkaline tolerance, whereas SNPs from the CK group were more closely related to plant growth under normal conditions rather than alkaline tolerance (Figure S2). Consequently, our subsequent study focused exclusively on the SNPs detected in these two groups, resulting in the identification of 28 significant SNPs associated with alkaline tolerance.

### 2.5. Quantitative Trait Loci Analysis

Based on the results of the GWAS, three significant SNPs—Chr01_38897254, Chr18_27591088, and Chr20_25660093—were identified across multiple GWAS models and groups (AT and AT/CK). By examining the upstream and downstream distances within the linkage disequilibrium (LD) decay (±71.6 kb) surrounding these significant SNPs, we delineated their associated genomic regions as stable QTLs, designated as *qAT1*, *qAT18*, and *qAT20*, respectively. Additionally, 15 significant SNPs associated with SDW in the AT group were identified within the ±71.6 kb LD decay region on chromosome 14; this region can also be considered a stable QTL associated with alkaline tolerance in soybean, referred to as *qAT14*. Moreover, four significant SNPs—Chr04_51929177, Chr04_52367033, Chr04_51934424, and Chr04_52131534—associated with SFW in the AT/CK group were identified within the ±71.6 kb LD decay on chromosome 4, across all seven GWAS models. Thus, this genomic region can also be delineated as a QTL, termed *qAT4*. Overall, we identified a total of five stable QTLs—*qAT1*, *qAT4*, *qAT14*, *qAT18*, and *qAT20*—associated with alkaline tolerance in soybean. Therefore, these SNPs are regarded as stable marker–trait associations (MTAs) that regulate alkaline tolerance in soybean.

### 2.6. Candidate Gene Identification

Based on the soybean reference genome, we identified a total of 39 genes within the physical intervals of five major QTLs (Table S4). Additionally, through gene annotations and literature surveys, we identified 17 candidate genes located within the genomic intervals of these five QTLs (Table S4). The selection of these candidate genes was guided by functional annotations related to various biological processes, including salt tolerance, drought tolerance, protein phosphorylation, heat shock proteins, basic helix–loop–helix (bHLH) transcription factors, heat shock transcription factors, S-ribonuclease binding protein 1, GRAS family transcription factors, pentatricopeptide repeat (PPR-like) superfamily proteins, C2H2-like zinc finger proteins, abiotic stress tolerance, auxin signaling, kinase activity, ethylene signaling, and salicylic acid signaling (Table 2). Each of these 17 genes exhibited at least one of these functions, thereby qualifying them as putative candidate genes regulating alkaline tolerance in soybean.

### 2.7. Haplotype Identification for Alkaline Tolerance

**Figure 4:**
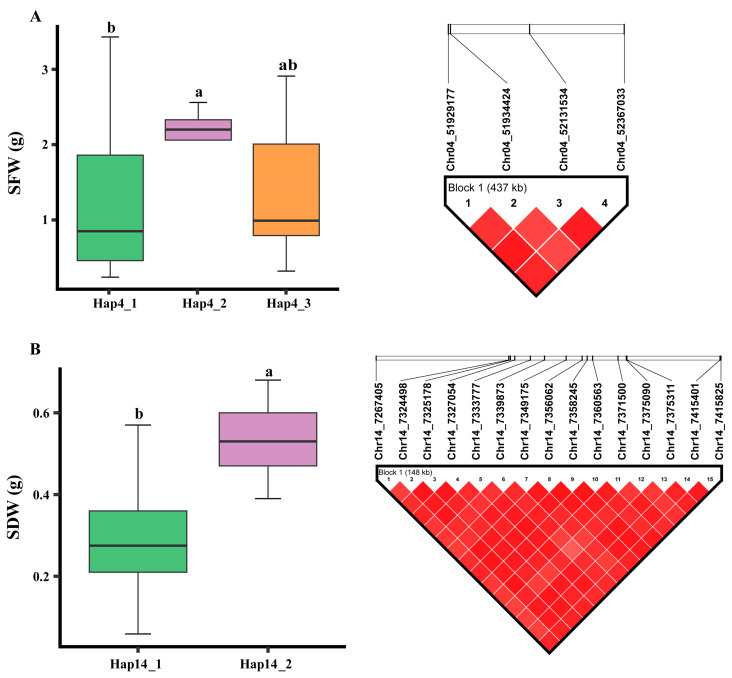
Haplotype allele analysis underlying two haplotype blocks designated as Hap4 (identified on Chr.04) and Hap14 (identified on Chr.14). (**A**) Phenotypic effects of different haplotype alleles of Hap4 block on SFW; (**B**) phenotypic effects of different haplotype alleles within Hap14 block on SDW. Grouping of genotypes and pairwise comparisons of genotypes were performed by using Bonferroni method at *p* < 0.05. Common letters above boxes indicate non-significant differences, while different letters denote significant differences.

Our results indicated that four significant SNPs are located on *qAT4*, associated with SFW in the AT/CK group. These SNPs formed a haplotype block designated as Hap4, which comprised three haplotype alleles: Hap4_1, Hap4_2, and Hap4_3 (Figure 4A). Hap4_2 was associated with higher SFW values in the AT/CK group, thus contributing to greater alkaline tolerance, while Hap4_1 was linked to the lowest SFW values and lower alkaline tolerance. Additionally, Hap4_3 was associated with intermediate SFW values, indicating moderate alkaline tolerance in soybean. Moreover, 15 significant SNPs on chromosome 14, associated with SDW in the AT group, formed another haplotype block designated as Hap14 (Figure 4B). This haplotype block comprised two haplotype alleles: Hap14_1 and Hap14_2 (Figure 4B). Hap14_1 was associated with the lowest SDW values in the AT group, correlating with lower alkaline tolerance in soybean, whereas Hap14_2 was linked to the highest SDW values, indicating higher alkaline tolerance.

**Figure 5:**
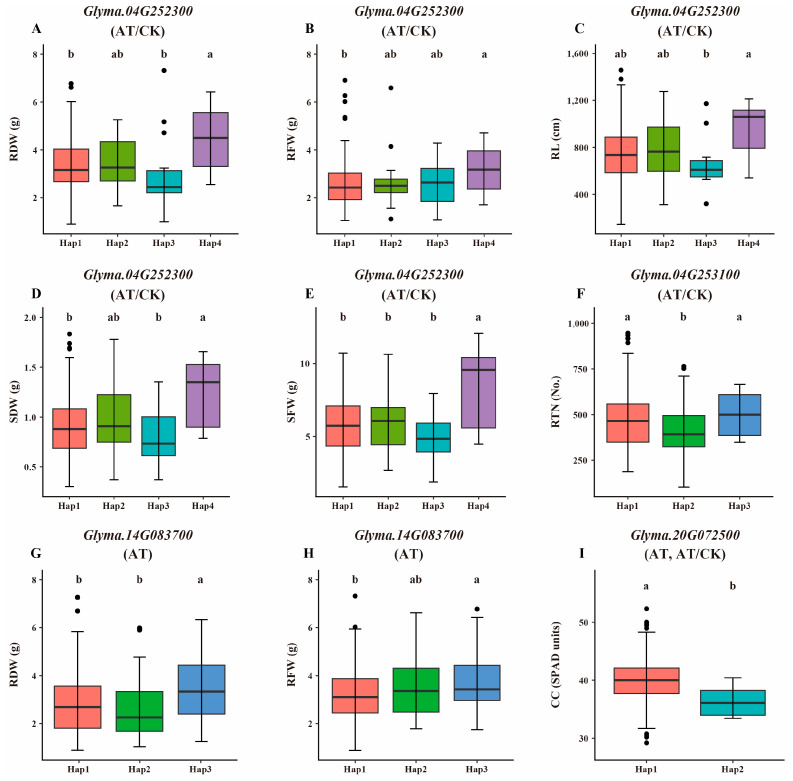
Haplotype analysis of candidate genes in GWAS panel of 326 soybean accessions. Phenotypic effects of different haplotype alleles of *Glyma.04G252300* (**A**–**E**), *Glyma.04G253100* (**F**), *Glyma.14G083700* (**G**,**H**) and *Glyma.20G072500* (**I**). Grouping of genotypes and pairwise comparisons of genotypes were performed by using Bonferroni method at *p* < 0.05. Different letters (a, b) indicate significant differences, while sharing same letter (e.g., a and ab) indicates non-significant differences. Dots placed beyond line edges indicate outliers, representing data points that deviate significantly from overall mean or fall outside typical range of variation in dataset.

Haplotype analysis of the 17 candidate genes revealed significant differences in the regulation of eight traits related to alkaline tolerance among the haplotype alleles of four specific genes: *Glyma.04G252300*, *Glyma.04G253100*, *Glyma.14G083700*, and *Glyma.20G072500*. The number of haplotype alleles associated with these genes across the GWAS panel of accessions ranged from two to four, exhibiting varied effects on alkaline tolerance. The phenotypic differences among the haplotype alleles in both AT and AT/CK groups are illustrated in Figure 5. In the AT/CK group, RDW, RFW, RL, SDW, and SFW exhibited significant differences among the haplotype alleles of *Glyma.04G252300*. Additionally, in the AT/CK group, RTN showed significant differences among the haplotype alleles of *Glyma.04G253100*. In the AT group, RDW and RFW displayed significant differences among the haplotype alleles of *Glyma.14G083700*, while CC exhibited significant differences among the haplotype alleles of *Glyma.20G072500* in both the AT and AT/CK groups. Interestingly, apart from *Glyma.04G253100*, the other three candidate genes exhibited significant haplotype differences among the same traits in the CK group (Figure S3).

## 3. Discussion

Recent advances in genome sequencing, genotyping platforms, and GWAS have enabled the high-resolution mapping of genetic loci and the identification of genes regulating specific traits [17]. In soybean, the GWAS approach has been employed to investigate the genetic basis of various traits [18]; however, research on the genetic basis of alkaline tolerance in soybean remains limited [16]. To address this gap, the present study utilized a high-throughput marker system within the GWAS framework to elucidate the genetic architecture of alkaline tolerance in soybean. Five novel QTLs related to alkaline tolerance were reported for the first time in this investigation.

Alkaline stress is frequently associated with salt stress, resulting from excessive alkaline salts such as NaHCO_3_ and Na_2_CO_3_ [19,20]. In our study, we identified 17 candidate genes associated with five QTLs, proposed as potential regulators of alkaline tolerance in soybean. Among these genes, the *Arabidopsis* orthologs of *Glyma.01G113400*, *Glyma.14G084500*, *Glyma.04G252100*, *Glyma.20G072600*, *Glyma.20G072700*, and *Glyma.20G072900* were annotated as being related to saline–alkaline stress (Table 2). The ortholog of *Glyma.04G252300* is *AtLAX3*, which plays a role in the inhibition of root elongation mediated by alkaline stress [21,22]. *Glyma.04G252700*, *Glyma.04G253100*, *Glyma.18G150300*, and *Glyma.20G072500* are linked to protein phosphorylation and kinase activity. The ortholog of Glyma.04G253100, PIP5K1 (phosphatidylinositol-4-phosphate 5-kinase 1), is induced by water stress and abscisic acid in *Arabidopsis* [23]. The ortholog of Glyma.20G072500, LECRK-S.7, is classified as a stress-related protein within the subfamily of legume lectin homologs [24]. Additionally, the ortholog of Glyma.14G083700 in *Arabidopsis* functions as a heat shock protein (HSP), which has been reported to confer tolerance to alkaline and salt stress in plants [25,26]. Glyma.04G251900 belongs to the GRAS family of proteins, which have been shown to regulate salt tolerance in *Melilotus albus* and kiwifruit [6,27]. Glyma.14G083900 functions as an E3 ubiquitin ligase, contributing to abiotic stress tolerance and negatively regulating responses to salt and alkaline stress [28]. Additionally, Glyma.04G252500 functions as a pentatricopeptide repeat protein, which has been reported to regulate salt and alkaline stress in *Arabidopsis* [29]. Therefore, the utilization of these genes in soybean breeding necessitates the prior verification of their functions through overexpression or knockout experiments. Following successful functional validation, these genes can then be employed in soybean breeding programs.

The research community has recognized the effectiveness of the GWAS approach in identifying genetic elements associated with complex traits. While SNP markers are biallelic, they are limited in their ability to detect multiple alleles, including rare and beneficial alleles related to crop traits in GWAS analyses [30]. In contrast, the multi-allelic nature of haplotype markers enables the capture of epistatic variation and rare or superior alleles present in diverse crop germplasm [31]. Superior haplotypes have been identified for various traits, such as grain quality in rice [32] and drought tolerance in pigeonpea [33]. Accurately detecting haplotypes for different crop traits, followed by their application in crop breeding, can significantly enhance genetic diversity in crop improvement efforts [34]. In this study, we identified haplotype alleles ranging from two to three within two detected haplotype blocks on chromosomes 4 and 14, associated with alkaline tolerance. Additionally, we identified haplotype alleles varying from two to four across four candidate genes: *Glyma.04G252300*, *Glyma.04G253100*, *Glyma.14G083700*, and *Glyma.20G072500*. These haplotype alleles correspond to varying phenotypes of alkaline tolerance, from low to high levels, providing an opportunity to enhance soybean alkaline tolerance. The incorporation of these haplotypes in soybean breeding programs will facilitate the development of alkaline-tolerant cultivars.

## 4. Materials and Methods

### 4.1. Plant Materials and Experimental Design

In the present study, we utilized a set of 326 diverse soybean germplasms collected from various agro-ecological regions of China for GWAS analysis (Table S2). These soybean germplasms were cultivated under controlled conditions using vermiculite in pots measuring 8 cm × 8 cm × 8 cm. The growth conditions were set to a photoperiod of 16 h of light and 8 h of darkness, with light-phase and dark-phase temperatures adjusted to 28 °C and 22 °C, respectively, and relative humidity maintained at 80%. A total of 18 pots were allocated for each soybean cultivar, with one seed sown per pot. Based on previous research [35], we selected a 150 mM concentration for alkaline treatment (NaHCO_3_: Na_2_CO_3_). After one week of germination, three pots with seedlings exhibiting similar growth vigor were selected and subjected to alkaline stress treatment (AT) using a Murashige and Skoog (MS) medium supplemented with NaHCO_3_ and Na_2_CO_3_ in a 5:1 ratio (pH 9.0 ± 0.1). The standard MS medium (pH 7.0 ± 0.1) served as the control (CK) for growing the soybean cultivars. For each treatment, three liters of the corresponding solution were added to a fixed tray containing the pots, and the solution was replaced every three days. After seven days, the seedlings from both the CK and AT treatments were used to measure phenotypic parameters. Three replications were conducted for each soybean accession, with three plants of each accession planted in each replication.

### 4.2. Phenotypic Data Analysis

We measured the phenotypic values of eight traits related to alkaline tolerance: shoot fresh weight (SFW), shoot dry weight (SDW), root dry weight (RDW), root fresh weight (RFW), chlorophyll content (CC), root number (RN), root tip number (RTN), and root length (RL). These traits were analyzed across three groups: CK, AT, and the ratio of AT to CK (AT/CK). The RN, RTN, and RL were measured using the RhizoPheno root analysis software (version 3.3) of the Intelligence Root Analysis System (Zhejiang Top Cloud-Agri root analyzer GXY-B, Hangzhou, China). The CC was estimated for the terminal leaflet of the first trifoliate leaf of soybean seedlings using the Konica Minolta SPAD-502Plus Portable Chlorophyll Meter (Soil and Plant Analyzer Development, SPAD, Tokyo, Japan) by following the default parameters as provided by the manufacturer. Measurements for CC were taken at the top, middle, and bottom of the leaflet, with the average of these three values considered the CC of the leaf. For the measurement of SFW and RFW, seedlings at seven days of AT were cut at the cotyledonary node to separate shoot and root tissues. A precision electric balance (Model JA203H, Changzhou Xingyun Electronic Equipment Co., Ltd., Changzhou, China) was used to estimate RFW and SFW for both CK and AT samples. These samples were then dried in an oven at 65 °C for five days to obtain the RDW and SDW using the same precision electric balance. The phenotypic values of these eight traits across three replications under control (CK), alkaline treatment (AT), and AT/CK conditions are presented in the Excel file of Table S5.

Data from individual groups were utilized to estimate the combined group data. The “lme4” package was employed for this estimation, following the methodology outlined by Di et al. [36]. Predicted means, known as Best Linear Unbiased Predictions (BLUPs), were calculated according to the method described by Agoyi et al. [37]. Descriptive statistical parameters for alkaline tolerance-related traits were estimated in accordance with the approach of El-Hashash [38].

### 4.3. Genotyping, Population Structure, and Linkage Disequilibrium (LD) Analysis

The association mapping panel, consisting of 326 accessions, was re-sequenced using the Illumina HiSeq platform (Novogene Company, Ltd., Beijing, China) at an average depth of 25×, resulting in a total data volume of 8.57 terabases (Tb). Adapter sequences, low-quality reads (where bases with a quality value of Q ≤ 20 accounted for over 50% of the read), and reads with more than 10% N content were removed using the fastp (v0.23.1) software [39], retaining only the clean reads. The alignment of the sequencing data with the reference genome *Wm82.a2.v1* was performed using BWA (v0.7.17) [40], and used the following parameter: “mem -t 10 -M -k 32 -R ‘@RG\tID:sampleID\tPL:ILLUMINA\tSM:sampleID’”. Variants, including single nucleotide polymorphisms (SNPs), insertions/deletions (InDels), structural variations (SVs), and copy number variations (CNVs), were identified and marked using the Genome Analysis Toolkit (GATK v4.2.6.1) [41]. Variant annotation was conducted by comparing each sample to the reference genome, with non-standard variants filtered out using quality control measures such as QD < 4.0, FS > 50.0, MQ < 40.0, and SOR > 3.0, resulting in 25,442,248 genetic variants. Further quality control measures, including a minor allele frequency (MAF) threshold of <0.05 and a missing genotype rate of 0.01, were applied to filter low-quality SNPs, yielding a total of 3,311,166 high-quality SNPs.

To estimate genome-wide LD, the PopLDdecay software (v3.41) [42] following the default options, viz., “-MaxDist 300”, was utilized to calculate the squared correlation coefficient (*r*^2^) between single nucleotide polymorphisms (SNPs) at known genomic positions. This software was also used to estimate the expected *r*^2^ under drift equilibrium and to visualize the results across physical distance (kilobases, kb). LD decay curves were fitted to scatter plots at the genomic level using smoothing spline regression lines [43], illustrating the relationship between LD and physical distance.

### 4.4. Genome-Wide Association Study Analysis

The GAPIT3 package [44] was utilized to conduct the GWAS. Seven GWAS models, GLM [45], MLM [46], CMLM [47], SUPER [48], FarmCPU [49], MLMM [50], and BLINK [51], fitted within the GAPIT3 package following the default commands, were employed in this study. The CLM, MLM, and CMLM models are single-locus models, while the remaining four are multi-locus models. To correct for population structure, the optimal number of principal component analyses (PCAs) was estimated using PLINK (v1.9) [52] following the command options, viz., plink --allow-extra-chr --threads 4 --bfile myfilename --pca 3 --out myfilename. The visualization of the population structure was performed in the R environment using the “ggplot2” package. Significant SNP associations were determined using the default parameters in GAPIT3, following the methodology outlined by Xu et al. [53].

### 4.5. Candidate Gene Analysis

For candidate gene identification, we downloaded all genes within the physical intervals of five QTLs from SoyBase (https://www.soybase.org/) using the Williams 82 (*Wm82.a2.v1*) gene model. The annotations for these genes were also retrieved from SoyBase. Based on gene function annotations and a literature search, candidate genes located within a physical interval of ±71.6 kb of the stable QTLs were selected [38].

### 4.6. Haplotype Analysis

Haploview 4.2 software was used to calculate the level of linkage disequilibrium (LD) among SNP pairs by following the default parameters [54]. The closest SNPs within the ±71.6 kb genomic interval formed the haplotype block [4]. The “confidence intervals” algorithm was employed to define the haplotype blocks [55]. Haplotype allele analysis and their phenotypic effect analysis were conducted using the Bonferroni method, as described previously [38,56].

## 5. Conclusions

In this study, we employed an integrated approach combining GWAS, QTL analysis, candidate gene analysis, and haplotype analysis to elucidate the genetic architecture of alkaline tolerance in soybean. Our investigation identified 28 significant SNPs and five novel QTLs associated with alkaline tolerance: *qAT1*, *qAT4*, *qAT14*, *qAT18*, and *qAT20*, which are reported for the first time. Haplotype analysis indicated that the alleles of four genes, *Glyma.04G252300*, *Glyma.04G253100*, *Glyma.14G083700,* and *Glyma.20G072500*, exhibited significant differences in their regulation of alkaline tolerance. The identified SNPs and QTLs will undergo further validation across multiple genetic backgrounds to facilitate their applications in soybean breeding.

## Figures and Tables

**Table 1:** Alkaline tolerance-related traits evaluated in 326 soybean accessions *.

Trait	Group	Min	Max	Median	Mean ± SD	CV%	Skewness	Kurtosis
RFW	CK	0.89	7.32	3.16	3.35 ± 1.09	32.54	0.56	0.28
	AT	0.15	3.47	1.28	1.36 ± 0.66	48.53	0.50	−0.31
	AT/CK	0.04	1.33	0.40	0.42 ± 0.20	47.62	0.98	1.57
RDW	CK	0.08	2.07	0.26	0.27 ± 0.13	48.15	7.98	106.01
	AT	0.01	0.31	0.10	0.10 ± 0.06	60.00	0.57	−0.31
	AT/CK	0.04	2.38	0.37	0.41 ± 0.26	63.41	2.24	10.48
SFW	CK	1.56	12.06	5.77	5.90 ± 1.95	33.05	0.49	−0.12
	AT	0.24	3.43	1.03	1.26 ± 0.82	65.08	0.51	−1.04
	AT/CK	0.04	1.04	0.18	0.22 ± 0.14	63.64	1.29	2.77
SDW	CK	0.3	1.83	0.88	0.91 ± 0.30	32.97	0.58	0.13
	AT	0.06	0.75	0.29	0.31 ± 0.12	38.71	0.71	0.04
	AT/CK	0.10	1.11	0.34	0.36 ± 0.15	41.67	1.34	3.33
RN	CK	406.33	4269.33	2030.50	2108.10 ± 718.17	34.07	0.29	−0.26
	AT	79.00	1666.33	505.16	587.59 ± 335.61	57.12	0.78	−0.08
	AT/CK	0.03	1.41	0.26	0.29 ± 0.17	58.62	1.86	6.58
RL	CK	143.85	1457.82	734.00	742.17 ± 227.12	30.60	0.25	−0.20
	AT	41.37	621.99	226.25	248.98 ± 121.32	48.73	0.59	−0.36
	AT/CK	0.04	1.52	0.32	0.35 ± 0.17	48.57	1.56	5.66
RTN	CK	103.33	947.00	434.83	449.18 ± 150.85	33.58	0.65	0.43
	AT	61.00	534.00	199.83	204.29 ± 77.06	37.72	0.55	0.33
	AT/CK	0.09	1.82	0.47	0.49 ± 0.22	44.90	1.35	4.54
CC	CK	29.22	52.33	39.98	39.95 ± 3.87	9.69	0.11	0.19
	AT	1.49	39.74	15.07	14.75 ± 10.17	68.95	0.20	−1.32
	AT/CK	0.04	0.98	0.38	0.36 ± 0.24	66.67	0.19	−1.30

* Min: minimum value; max: maximum value; SD: standard deviation; CV: coefficient of variation; CK: control; AT: alkaline stress treatment; AT/CK: the ratio of the trait value under AT compared to CK.

**Table 2:** Candidate genes underlying the identified QTLs.

No.	Gene ID	*Arabidopsis* Ortholog	Gene Function Annotation
1	*Glyma.01G113400*	*AT4G00430* (plasma membrane intrinsic protein 1B)	Response to salt stress, response to temperature stimulus, response to water deprivation and water transport
2	*Glyma.04G251900*	*AT4G08250* (GRAS family transcription factor)	Regulation of transcription, DNA-dependent
3	*Glyma.04G252100*	*AT4G36020* (cold shock domain protein 1)	Response to cold, response to salt stress and response to water deprivation
4	*Glyma.04G252300*	*AT1G77690* (an auxin influx carrier LAX3)	Response to UV light, auxin polar transport, brassinosteroid biosynthetic process, response to auxin stimulus, response to cyclopentenone, root cap development and root hair elongation.
5	*Glyma.04G252500*	*AT4G08210* (Pentatricopeptide repeat (PPR-like) superfamily protein)	Biological process
6	*Glyma.04G252600*	*AT1G75710* (C2H2-like zinc finger protein)	NA
7	*Glyma.04G252700*	*AT1G77720* (PPK1, putative protein kinase 1)	DNA methylation, protein autophosphorylation, and protein phosphorylation
8	*Glyma.04G253000*	*AT4G08180* (OSBP (oxysterol binding protein)-related protein 1C)	Abscisic acid-mediated signaling pathway, response to cold, response to ethylene stimulus, and systemic acquired resistance
9	*Glyma.04G253100*	*AT1G21980* (PIP5K1, phosphatidylinositol-4-phosphate 5-kinase 1)	Phosphatidylinositol metabolic process
10	*Glyma.14G083700*	*AT1G46264* (AtHSFB4, heat shock transcription factor B4)	Response to abiotic stress and response to heat
11	*Glyma.14G083900*	*AT1G45976* (S-ribonuclease binding protein 1)	Biological process; hormone-mediated signaling pathway; photoperiodism, flowering; signal transduction
12	*Glyma.14G084500*	*AT4G34110* (poly(A) binding protein 2)	Response to salt stress
13	*Glyma.18G150300*	*AT5G10530* (Concanavalin A-like lectin protein kinase family protein)	Protein phosphorylation
14	*Glyma.20G072500*	*AT5G55830* (LECRK-S.7, L-type lecting receptor kinase S.7)	Protein phosphorylation
15	*Glyma.20G072600*	*AT5G03540* (exocyst subunit exo70 family protein A1)	Auxin transport, hyperosmotic response, protein localization involved in auxin polar transport, response to salt stress, response to temperature stimulus, root development, and root hair elongation
16	*Glyma.20G072700*	*AT5G03540* (exocyst subunit exo70 family protein A1)	Auxin transport, hyperosmotic response, protein localization involved in auxin polar transport, response to salt stress, response to temperature stimulus, root development, and root hair elongation
17	*Glyma.20G072900*	*AT5G03540* (exocyst subunit exo70 family protein A1)	Auxin transport, hyperosmotic response, protein localization involved in auxin polar transport, response to salt stress, response to temperature stimulus, root development, and root hair elongation

## Data Availability

Resequencing data generated in this study have been deposited in the VCF format at the Genome Variation Map (GVM) database in the BIG Data Center (https://ngdc.cncb.ac.cn/gvm/submit/sub001785/accession (accessed on 15 January 2025)) under the accession number GVM000945.

## Supplementary Materials

The following supporting information can be downloaded at https://www.mdpi.com/article/10.3390/plants14030357/s1, Figure S1: Pearson correlation coefficient among the related traits. Figure S2: GWAS analysis of related traits in CK group. Figure S3: Haplotype analysis of candidate genes in CK group. Table S1: Distribution of all the SNPs across the 20 soybean chromosomes. Table S2: The 326 soybean accessions used in the GWAS analysis. Table S3: Significant SNP markers associated with related traits. Table S4: The position of gene underlying the QTLs. Table S5: The phenotypic information of eight traits of 326 soybean accessions.
