# Supplementary material for: Deciphering of Genomic Loci Associated with Alkaline Tolerance in Soybean [Glycine max (L.) Merr.] by Genome-Wide Association Study
Source: Plants (Basel). 2025 Jan 24;14(3):357. doi: 10.3390/plants14030357 (PMC11820895; doi:10.3390/plants14030357)
Supplement: Supplementary file 1 [file plants-14-00357-s001.zip › Yang-Supp-0116(2).pdf]

Supplementary Materials:

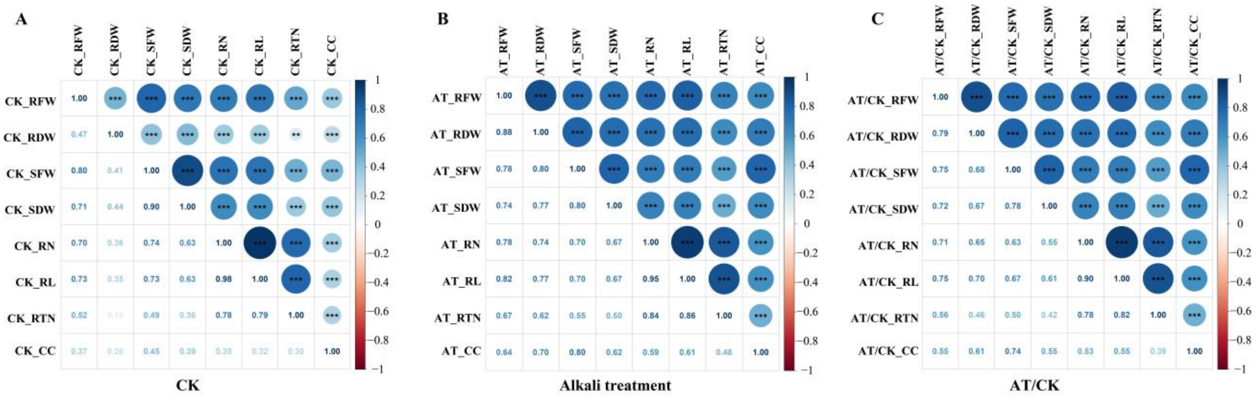

Figure S1. Pearson correlation coefficient among the related traits. These traits include root fresh weight (RFW), root dry weight (RDW), seedling fresh weight (SFW), seedling dry weight (SDW), root number (RN), root length (RL), root tips number (RTN) and relative chlorophyll content (CC). The analysis was conducted across three groups: (A) CK group; (B) AT group; (C) AT/CK group.

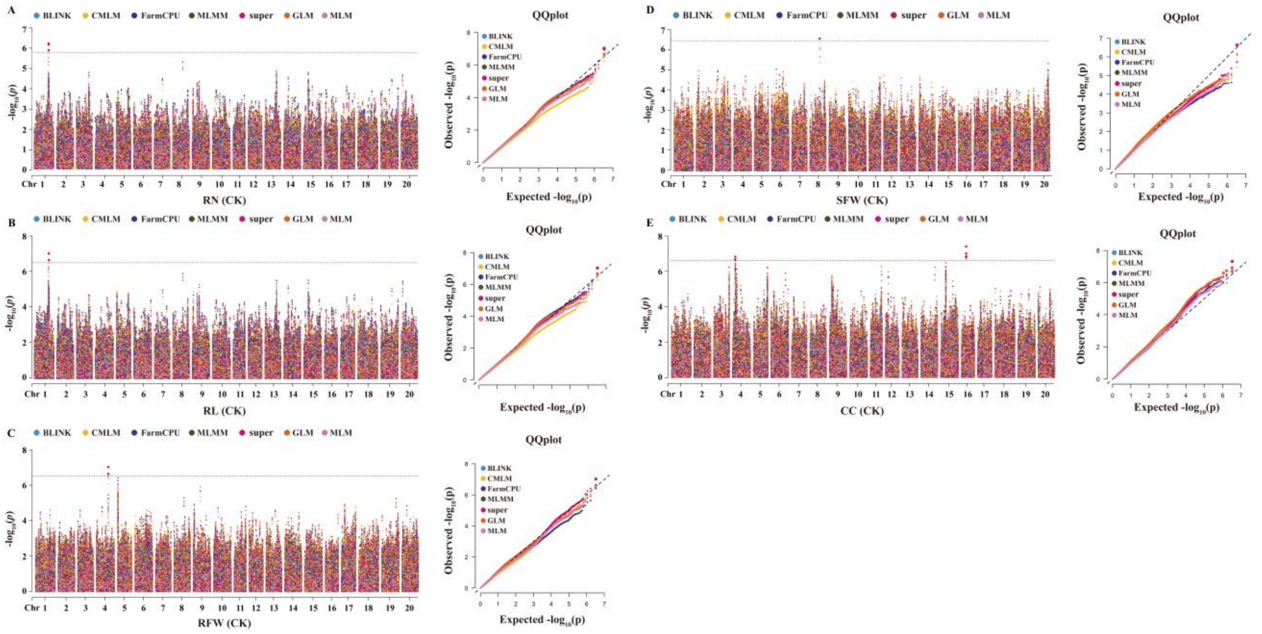

Figure S2. **GWAS analysis of related traits in CK group.** (A-E) Manhattan and QQ plot for RN, RL, RFW, SFW and CC in the CK group, respectively. The horizontal dotted black line represents the threshold level of significance ($-\log_{10}P$> 6.67), and the soybean chromosomes are represented on the X-axis.

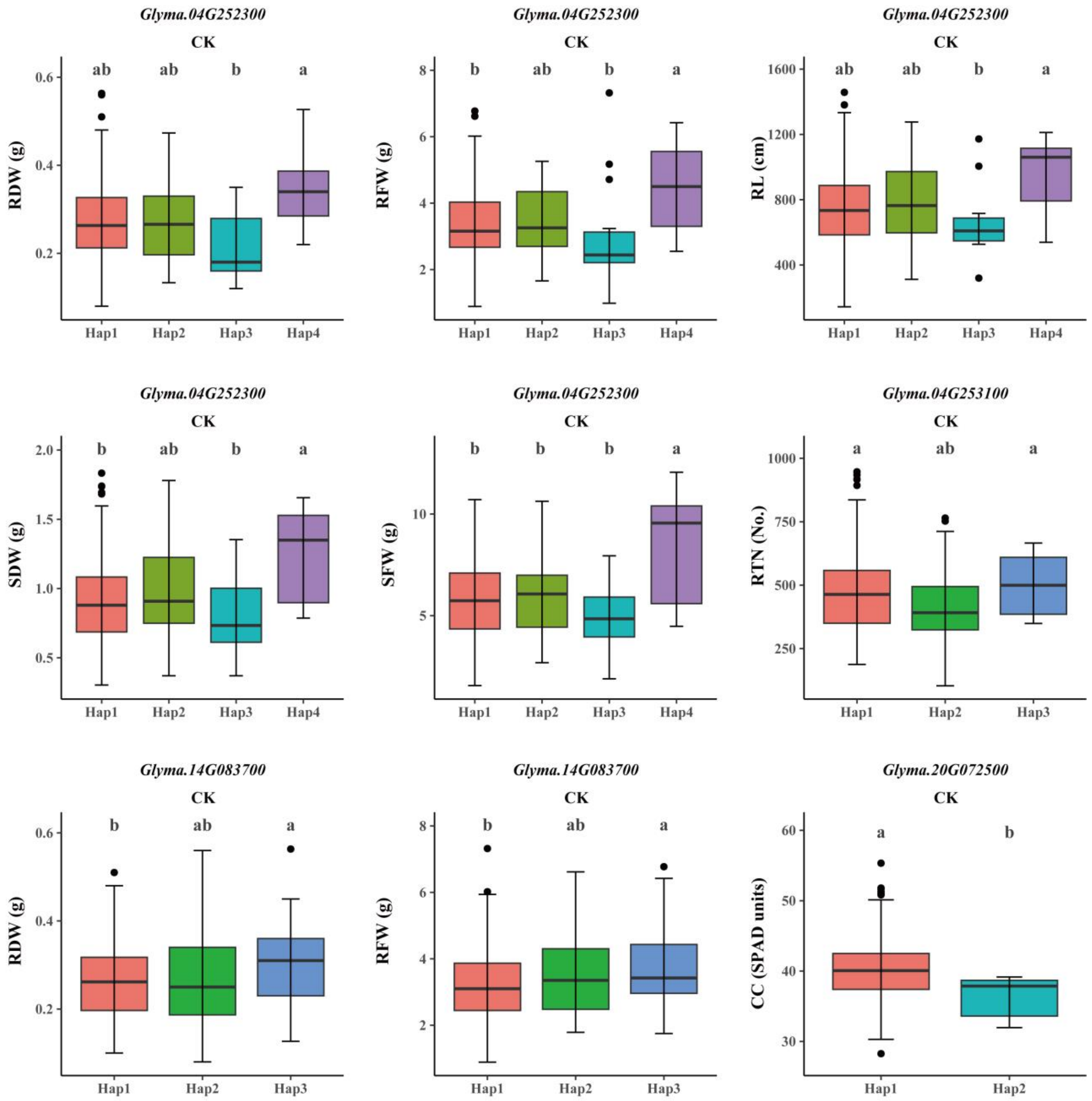

Figure S3. **Haplotype analysis of candidate genes in CK group.** RDW: root dry weight; RFW: root fresh weight; RL: total root length; SDW: seedling dry weight; SFW: seedling fresh weight; RTN: root tips number; CC: relative chlorophyll content; Grouping of genotypes and pairwise comparisons of genotypes were performed by using Bonferroni method at $P < 0.05$. Different letters (a, b) indicate significant differences while sharing the same letter (e.g., a and ab) indicates no significant differences. Dots outside the whiskers represent outliers, which are data points that deviate significantly from the overall mean or fall beyond the typical range of variation in the dataset.

Table S1. Distribution of all the SNPs across the 20 soybean chromosomes

Chromosome	Length (bp)	Length (Mb)	Numbers of SNPs	Average Inter-marker distance (bp)	Density (SNPs/Mb)
Chr01	56,831,624	56.83	145,161	391.51	2554.30
Chr02	48,577,505	48.58	137,295	353.82	2826.16
Chr03	45,779,781	45.78	197,800	231.44	4320.66
Chr04	52,389,146	52.39	212,335	246.73	4052.97
Chr05	42,234,498	42.23	88,978	474.66	2106.99
Chr06	51,416,486	51.42	207,373	247.94	4032.92
Chr07	44,630,646	44.63	137,170	325.37	3073.49
Chr08	47,837,940	47.84	126,937	376.86	2653.37
Chr09	50,189,764	50.19	199,014	252.19	3965.21
Chr10	51,566,898	51.57	153,297	336.39	2972.60
Chr11	34,766,867	34.77	62,554	555.79	1799.08
Chr12	40,091,314	40.09	98,765	405.93	2463.58
Chr13	45,874,162	45.87	169,154	271.20	3687.68
Chr14	49,042,192	49.04	136,485	359.32	2783.14
Chr15	51,756,343	51.76	254,495	203.37	4916.83
Chr16	37,887,014	37.89	175,982	215.29	4644.55
Chr17	41,641,366	41.64	177,023	235.23	4251.27
Chr18	58,018,742	58.02	250,124	231.96	4311.00
Chr19	50,746,916	50.75	215,581	235.40	4247.90
Chr20	47,904,181	47.90	165,643	289.20	3458.10
Total	949,183,385	949.19	3,311,166	286.66	3488.41

Table S2. The 326 soybean accessions used in the GWAS analysis*

No.	Name	Group	Region	No.	Name	Group	Region
A001	FENGGWS004	I	Harbing,Heilongjiang, China	A061	FENGGWS067	II	Jilin,Jilin, China
A002	FENGGWS005	I	Harbing,Heilongjiang, China	A062	FENGGWS068	II	Jilin,Jilin, China
A003	FENGGWS006	I	Harbing,Heilongjiang, China	A063	FENGGWS069	II	Jilin,Jilin, China
A004	FENGGWS007	I	Harbing,Heilongjiang, China	A064	FENGGWS070	II	Jilin,Jilin, China
A005	FENGGWS008	I	Harbing,Heilongjiang, China	A065	FENGGWS071	II	Jilin,Jilin, China
A006	FENGGWS009	I	Keshan,Heilongjiang, China	A066	FENGGWS072	II	Jilin,Jilin, China
A007	FENGGWS010	I	Keshan,Heilongjiang, China	A067	FENGGWS073	II	Jilin,Jilin, China
A008	FENGGWS011	I	Keshan,Heilongjiang, China	A068	FENGGWS074	VI	Kunming,Yunnan, China
A009	FENGGWS012	I	Keshan,Heilongjiang, China	A069	FENGGWS075	I	Harbing,Heilongjiang, China
A010	FENGGWS013	I	Keshan,Heilongjiang, China	A070	FENGGWS076	V	Bengbu,Anhui, China
A011	FENGGWS014	I	Keshan,Heilongjiang, China	A071	FENGGWS077	II	Jilin,Jilin, China
A012	FENGGWS015	I	Keshan,Heilongjiang, China	A072	FENGGWS078	III	Dandong,Liaoning, China
A013	FENGGWS016	I	Keshan,Heilongjiang, China	A073	FENGGWS079	III	Dandong,Liaoning, China
A014	FENGGWS017	I	Heihe,Heilongjiang, China	A074	FENGGWS080	II	Gongzhuling,Jilin, China
A015	FENGGWS018	I	Heihe,Heilongjiang, China	A075	FENGGWS081	I	Shangzhi,Heilongjiang, China
A016	FENGGWS019	I	Heihe,Heilongjiang, China	A076	FENGGWS082	II	Gongzhuling,Jilin, China
A017	FENGGWS020	I	Heihe,Heilongjiang, China	A077	FENGGWS083	II	Gongzhuling,Jilin, China
A018	FENGGWS021	I	Jiamusi,Heilongjiang, China	A078	FENGGWS084	I	Harbing,Heilongjiang, China
A019	FENGGWS022	I	Jiamusi,Heilongjiang, China	A079	FENGGWS085	I	Harbing,Heilongjiang, China
A020	FENGGWS023	I	Jiamusi,Heilongjiang, China	A080	FENGGWS086	I	Harbing,Heilongjiang, China
A021	FENGGWS024	I	Jiamusi,Heilongjiang, China	A081	FENGGWS087	II	Gongzhuling,Jilin, China
A022	FENGGWS025	I	Jiamusi,Heilongjiang, China	A082	FENGGWS088	II	Gongzhuling,Jilin, China
A023	FENGGWS026	I	Jiamusi,Heilongjiang, China	A083	FENGGWS089	II	Gongzhuling,Jilin, China
A024	FENGGWS027	I	Jiamusi,Heilongjiang, China	A084	FENGGWS090	I	Gongzhuling,Heilongjiang, China
A025	FENGGWS028	I	Jiamusi,Heilongjiang, China	A085	FENGGWS091	I	Gongzhuling,Heilongjiang, China
A026	FENGGWS029	I	Mudanjiang,Heilongjiang, China	A086	FENGGWS092	I	Gongzhuling,Heilongjiang, China
A027	FENGGWS030	I	Mudanjiang,Heilongjiang, China	A087	FENGGWS093	II	Gongzhuling,Jilin, China
A028	FENGGWS031	I	Mudanjiang,Heilongjiang, China	A088	FENGGWS094	II	Gongzhuling,Jilin, China
A029	FENGGWS032	I	Qiqihar,Heilongjiang, China	A089	FENGGWS095	II	Gongzhuling,Jilin, China
A030	FENGGWS033	I	Suihua,Heilongjiang, China	A090	FENGGWS096	II	Gongzhuling,Jilin, China
A031	FENGGWS034	I	Suihua,Heilongjiang, China	A091	FENGGWS097	II	Gongzhuling,Jilin, China
A032	FENGGWS035	II	Gongzhuling,Jilin, China	A092	FENGGWS098	II	Gongzhuling,Jilin, China
A033	FENGGWS036	I	Harbing,Heilongjiang, China	A093	FENGGWS099	II	Gongzhuling,Jilin, China
A034	FENGGWS037	I	Harbing,Heilongjiang, China	A094	FENGGWS100	II	Gongzhuling,Jilin, China
A035	FENGGWS038	I	Keshan,Heilongjiang, China	A095	FENGGWS101	II	Gongzhuling,Jilin, China
A036	FENGGWS039	I	Suihua,Heilongjiang, China	A096	FENGGWS102	II	Gongzhuling,Jilin, China
A037	FENGGWS040	I	Suihua,Heilongjiang, China	A097	FENGGWS103	III	Dandong,Liaoning, China
A038	FENGGWS041	I	Suihua,Heilongjiang, China	A098	FENGGWS104	III	Dandong,Liaoning, China
A039	FENGGWS042	I	Harbing,Heilongjiang, China	A099	FENGGWS105	II	Gongzhuling,Jilin, China
A040	FENGGWS043	II	Gongzhuling,Jilin, China	A100	FENGGWS106	II	Gongzhuling,Jilin, China
A041	FENGGWS044	I	Suihua,Heilongjiang, China	A101	FENGGWS107	II	Gongzhuling,Jilin, China
A042	FENGGWS045	I	Harbing,Heilongjiang, China	A102	FENGGWS108	II	Gongzhuling,Jilin, China
A043	FENGGWS047	I	Harbing,Heilongjiang, China	A103	FENGGWS110	II	Gongzhuling,Jilin, China
A044	FENGGWS048	I	Harbing,Heilongjiang, China	A104	FENGGWS111	II	Gongzhuling,Jilin, China
A045	FENGGWS049	I	Gongzhuling,Heilongjiang, China	A105	FENGGWS112	II	Gongzhuling,Jilin, China
A046	FENGGWS050	I	Keshan,Heilongjiang, China	A106	FENGGWS113	II	Gongzhuling,Jilin, China
A047	FENGGWS051	III	Dandong,Liaoning, China	A107	FENGGWS114	II	Gongzhuling,Jilin, China
A048	FENGGWS052	I	Harbing,Heilongjiang, China	A108	FENGGWS115	II	Gongzhuling,Jilin, China
A049	FENGGWS053	I	Suihua,Heilongjiang, China	A109	FENGGWS116	I	Harbing,Heilongjiang, China
A050	FENGGWS054	I	Jiamusi,Heilongjiang, China	A110	FENGGWS117	II	Gongzhuling,Jilin, China
A051	FENGGWS055	I	Jiamusi,Heilongjiang, China	A111	FENGGWS118	II	Hunchun,Jilin, China
A052	FENGGWS056	I	Jiamusi,Heilongjiang, China	A112	FENGGWS119	II	Gongzhuling,Jilin, China
A053	FENGGWS057	II	Gongzhuling,Jilin, China	A113	FENGGWS120	II	Gongzhuling,Jilin, China
A054	FENGGWS059	II	Gongzhuling,Jilin, China	A114	FENGGWS121	II	Gongzhuling,Jilin, China
A055	FENGGWS061	II	Gongzhuling,Jilin, China	A115	FENGGWS122	III	Dandong,Liaoning, China
A056	FENGGWS062	II	Gongzhuling,Jilin, China	A116	FENGGWS123	II	Gongzhuling,Jilin, China
A057	FENGGWS063	II	Gongzhuling,Jilin, China	A117	FENGGWS124	II	Gongzhuling,Jilin, China
A058	FENGGWS064	II	Gongzhuling,Jilin, China	A118	FENGGWS125	V	Taiyuan,Shanxi, China
A059	FENGGWS065	II	Gongzhuling,Jilin, China	A119	FENGGWS126	VI	Kunming,Yunnan, China
A060	FENGGWS066	II	Gongzhuling,Jilin, China	A120	FENGGWS127	II	Gongzhuling,Jilin, China

No.	Name	Group	Region	No.	Name	Group	Region
A121	FENGGWS128	II	Gongzhuling,Jilin, China	A181	FENGGWS191	I	Harbing,Heilongjiang, China
A122	FENGGWS129	II	Gongzhuling,Jilin, China	A182	FENGGWS194	I	Harbing,Heilongjiang, China
A123	FENGGWS131	III	Dandong,Liaoning, China	A183	FENGGWS195	I	Harbing,Heilongjiang, China
A124	FENGGWS132	I	Harbing,Heilongjiang, China	A184	FENGGWS196	I	Harbing,Heilongjiang, China
A125	FENGGWS133	II	Gongzhuling,Jilin, China	A185	FENGGWS197	I	Harbing,Heilongjiang, China
A126	FENGGWS134	V	Tangshan,Hebei, China	A186	FENGGWS198	I	Suihua,Heilongjiang, China
A127	FENGGWS135	II	Gongzhuling,Jilin, China	A187	FENGGWS200	I	Harbing,Heilongjiang, China
A128	FENGGWS136	II	Gongzhuling,Jilin, China	A188	FENGGWS201	I	Harbing,Heilongjiang, China
A129	FENGGWS138	III	Tieling,Liaoning, China	A189	FENGGWS202	I	Harbing,Heilongjiang, China
A130	FENGGWS139	III	Tieling,Liaoning, China	A190	FENGGWS204	II	Gongzhuling,Jilin, China
A131	FENGGWS140	III	Tieling,Liaoning, China	A191	FENGGWS205	I	Harbing,Heilongjiang, China
A132	FENGGWS141	III	Tieling,Liaoning, China	A192	FENGGWS206	II	Gongzhuling,Jilin, China
A133	FENGGWS142	III	Tieling,Liaoning, China	A193	FENGGWS207	I	Harbing,Heilongjiang, China
A134	FENGGWS143	III	Tieling,Liaoning, China	A194	FENGGWS208	III	Dandong,Liaoning, China
A135	FENGGWS144	III	Tieling,Liaoning, China	A195	FENGGWS209	I	Harbing,Heilongjiang, China
A136	FENGGWS145	III	Dandong,Liaoning, China	A196	FENGGWS210	I	Harbing,Heilongjiang, China
A137	FENGGWS146	III	Dandong,Liaoning, China	A197	FENGGWS211	I	Harbing,Heilongjiang, China
A138	FENGGWS147	III	Dandong,Liaoning, China	A198	FENGGWS212	I	Harbing,Heilongjiang, China
A139	FENGGWS148	III	Dandong,Liaoning, China	A199	FENGGWS213	I	Harbing,Heilongjiang, China
A140	FENGGWS149	I	Harbing,Heilongjiang, China	A200	FENGGWS214	I	Harbing,Heilongjiang, China
A141	FENGGWS150	II	Gongzhuling,Jilin, China	A201	FENGGWS215	II	Gongzhuling,Jilin, China
A142	FENGGWS151	V	Huaibei,Anhui, China	A202	FENGGWS216	II	Gongzhuling,Jilin, China
A143	FENGGWS152	I	Harbing,Heilongjiang, China	A203	FENGGWS217	I	Harbing,Heilongjiang, China
A144	FENGGWS153	II	Gongzhuling,Jilin, China	A204	FENGGWS218	I	Harbing,Heilongjiang, China
A145	FENGGWS154	VI	Chengdu,Sichuan, China	A205	FENGGWS219	I	Harbing,Heilongjiang, China
A146	FENGGWS155	III	Dandong,Liaoning, China	A206	FENGGWS220	I	Harbing,Heilongjiang, China
A147	FENGGWS156	I	Harbing,Heilongjiang, China	A207	FENGGWS221	I	Harbing,Heilongjiang, China
A148	FENGGWS157	II	Gongzhuling,Jilin, China	A208	FENGGWS222	I	Harbing,Heilongjiang, China
A149	FENGGWS158	VI	Chengdu,Sichuan, China	A209	FENGGWS223	II	Gongzhuling,Jilin, China
A150	FENGGWS159	I	Harbing,Heilongjiang, China	A210	FENGGWS224	I	Harbing,Heilongjiang, China
A151	FENGGWS160	III	Dandong,Liaoning, China	A211	FENGGWS225	III	Dandong,Liaoning, China
A152	FENGGWS161	I	Harbing,Heilongjiang, China	A212	FENGGWS226	I	Harbing,Heilongjiang, China
A153	FENGGWS162	I	Harbing,Heilongjiang, China	A213	FENGGWS227	II	Gongzhuling,Jilin, China
A154	FENGGWS163	II	Gongzhuling,Jilin, China	A214	FENGGWS228	V	Tangshan,Hebei, China
A155	FENGGWS164	III	Dandong,Liaoning, China	A215	FENGGWS229	I	Harbing,Heilongjiang, China
A156	FENGGWS165	I	Harbing,Heilongjiang, China	A216	FENGGWS230	III	Dandong,Liaoning, China
A157	FENGGWS166	III	Dandong,Liaoning, China	A217	FENGGWS231	I	Harbing,Heilongjiang, China
A158	FENGGWS167	III	Dandong,Liaoning, China	A218	FENGGWS232	III	Dandong,Liaoning, China
A159	FENGGWS168	III	Tieling,Liaoning, China	A219	FENGGWS233	I	Harbing,Heilongjiang, China
A160	FENGGWS169	III	Dandong,Liaoning, China	A220	FENGGWS234	I	Harbing,Heilongjiang, China
A161	FENGGWS170	III	Dandong,Liaoning, China	A221	FENGGWS235	I	Harbing,Heilongjiang, China
A162	FENGGWS171	I	Harbing,Heilongjiang, China	A222	FENGGWS236	I	Harbing,Heilongjiang, China
A163	FENGGWS172	I	Harbing,Heilongjiang, China	A223	FENGGWS237	I	Harbing,Heilongjiang, China
A164	FENGGWS173	III	Dandong,Liaoning, China	A224	FENGGWS238	II	Gongzhuling,Jilin, China
A165	FENGGWS174	II	Gongzhuling,Jilin, China	A225	FENGGWS239	II	Gongzhuling,Jilin, China
A166	FENGGWS175	III	Dandong,Liaoning, China	A226	FENGGWS240	II	Gongzhuling,Jilin, China
A167	FENGGWS176	II	Gongzhuling,Jilin, China	A227	FENGGWS241	II	Gongzhuling,Jilin, China
A168	FENGGWS178	III	Tieling,Liaoning, China	A228	FENGGWS242	II	Gongzhuling,Jilin, China
A169	FENGGWS179	III	Dandong,Liaoning, China	A229	FENGGWS243	II	Gongzhuling,Jilin, China
A170	FENGGWS180	III	Dandong,Liaoning, China	A230	FENGGWS244	II	Gongzhuling,Jilin, China
A171	FENGGWS181	III	Tieling,Liaoning, China	A231	FENGGWS245	II	Gongzhuling,Jilin, China
A172	FENGGWS182	III	Dandong,Liaoning, China	A232	FENGGWS246	II	Gongzhuling,Jilin, China
A173	FENGGWS183	III	Dandong,Liaoning, China	A233	FENGGWS247	II	Tonghua,Jilin, China
A174	FENGGWS184	III	Tieling,Liaoning, China	A234	FENGGWS248	II	Tonghua,Jilin, China
A175	FENGGWS185	III	Tieling,Liaoning, China	A235	FENGGWS249	II	Tonghua,Jilin, China
A176	FENGGWS186	III	Dandong,Liaoning, China	A236	FENGGWS250	II	Tonghua,Jilin, China
A177	FENGGWS187	III	Dandong,Liaoning, China	A237	FENGGWS251	II	Gongzhuling,Jilin, China
A178	FENGGWS188	III	Dandong,Liaoning, China	A238	FENGGWS252	I	Harbing,Heilongjiang, China
A179	FENGGWS189	I	Harbing,Heilongjiang, China	A239	FENGGWS253	II	Gongzhuling,Jilin, China
A180	FENGGWS190	I	Harbing,Heilongjiang, China	A240	FENGGWS254	II	Gongzhuling,Jilin, China

No.	Name	Group	Region	No.	Name	Group	Region
A241	FENGGWS255	II	Gongzhuling,Jilin, China	A284	FENGGWS320	III	Tieling,Liaoning, China
A242	FENGGWS257	II	Gongzhuling,Jilin, China	A285	FENGGWS321	III	Dandong,Liaoning, China
A243	FENGGWS258	II	Gongzhuling,Jilin, China	A286	FENGGWS323	III	Tieling,Liaoning, China
A244	FENGGWS259	II	Gongzhuling,Jilin, China	A287	FENGGWS324	III	Tieling,Liaoning, China
A245	FENGGWS260	II	Gongzhuling,Jilin, China	A288	FENGGWS325	III	Dandong,Liaoning, China
A246	FENGGWS261	II	Gongzhuling,Jilin, China	A289	FENGGWS326	III	Dandong,Liaoning, China
A247	FENGGWS262	II	Gongzhuling,Jilin, China	A290	FENGGWS327	I	Harbing,Heilongjiang, China
A248	FENGGWS263	II	Jilin,Jilin, China	A291	FENGGWS329	V	Beijing,Beijing, China
A249	FENGGWS264	II	Gongzhuling,Jilin, China	A292	FENGGWS330	I	Harbing,Heilongjiang, China
A250	FENGGWS265	II	Gongzhuling,Jilin, China	A293	FENGGWS331	I	Harbing,Heilongjiang, China
A251	FENGGWS266	II	Gongzhuling,Jilin, China	A294	FENGGWS332	I	Harbing,Heilongjiang, China
A252	FENGGWS267	II	Gongzhuling,Jilin, China	A295	FENGGWS334	I	Harbing,Heilongjiang, China
A253	FENGGWS268	II	Gongzhuling,Jilin, China	A296	FENGGWS336	I	Harbing,Heilongjiang, China
A254	FENGGWS269	II	Gongzhuling,Jilin, China	A297	FENGGWS337	I	Suihua,Heilongjiang, China
A255	FENGGWS270	II	Gongzhuling,Jilin, China	A298	FENGGWS338	I	Suihua,Heilongjiang, China
A256	FENGGWS271	II	Gongzhuling,Jilin, China	A299	FENGGWS339	I	Harbing,Heilongjiang, China
A257	FENGGWS272	II	Gongzhuling,Jilin, China	A300	FENGGWS340	I	Harbing,Heilongjiang, China
A258	FENGGWS273	II	Gongzhuling,Jilin, China	A301	FENGGWS341	I	Harbing,Heilongjiang, China
A259	FENGGWS274	II	Gongzhuling,Jilin, China	A302	FENGGWS343	I	Harbing,Heilongjiang, China
A260	FENGGWS276	II	Gongzhuling,Jilin, China	A303	FENGGWS344	II	Gongzhuling,Jilin, China
A261	FENGGWS277	II	Gongzhuling,Jilin, China	A304	FENGGWS345	III	Dandong,Liaoning, China
A262	FENGGWS278	II	Gongzhuling,Jilin, China	A305	FENGGWS346	II	Gongzhuling,Jilin, China
A263	FENGGWS279	II	Gongzhuling,Jilin, China	A306	FENGGWS347	II	Gongzhuling,Jilin, China
A264	FENGGWS280	II	Gongzhuling,Jilin, China	A307	FENGGWS348	II	Gongzhuling,Jilin, China
A265	FENGGWS281	II	Gongzhuling,Jilin, China	A308	FENGGWS349	II	Tonghua,Jilin, China
A266	FENGGWS282	II	Gongzhuling,Jilin, China	A309	FENGGWS350	II	Tonghua,Jilin, China
A267	FENGGWS283	II	Hunchun,Jilin, China	A310	FENGGWS320	II	Gongzhuling,Jilin, China
A268	FENGGWS284	II	Gongzhuling,Jilin, China	A311	FENGGWS321	I	Harbing,Heilongjiang, China
A269	FENGGWS285	II	Gongzhuling,Jilin, China	A312	FENGGWS323	III	Dandong,Liaoning, China
A270	FENGGWS286	II	Tonghua,Jilin, China	A313	FENGGWS324	IV	Chifeng,Neimenggu, China
A271	FENGGWS287	II	Tonghua,Jilin, China	A314	FENGGWS325	IV	Chifeng,Neimenggu, China
A272	FENGGWS288	II	Gongzhuling,Jilin, China	A315	FENGGWS326	IV	Chifeng,Neimenggu, China
A273	FENGGWS289	II	Gongzhuling,Jilin, China	A316	FENGGWS327	V	Taiyuan,Shanxi, China
A274	FENGGWS290	II	Gongzhuling,Jilin, China	A317	FENGGWS329	IV	Chifeng,Neimenggu, China
A275	FENGGWS291	II	Gongzhuling,Jilin, China	A318	FENGGWS330	I	Harbing,Heilongjiang, China
A276	FENGGWS292	II	Gongzhuling,Jilin, China	A319	FENGGWS331	I	Harbing,Heilongjiang, China
A277	FENGGWS293	II	Gongzhuling,Jilin, China	A320	FENGGWS332	I	Harbing,Heilongjiang, China
A278	FENGGWS294	II	Gongzhuling,Jilin, China	A321	FENGGWS334	I	Harbing,Heilongjiang, China
A279	FENGGWS295	II	Gongzhuling,Jilin, China	A322	FENGGWS336	II	Gongzhuling,Jilin, China
A280	FENGGWS296	II	Gongzhuling,Jilin, China	A323	FENGGWS337	II	Gongzhuling,Jilin, China
A281	FENGGWS297	II	Gongzhuling,Jilin, China	A324	FENGGWS338	II	Jilin,Jilin, China
A282	FENGGWS298	II	Gongzhuling,Jilin, China	A325	FENGGWS339	II	Gongzhuling,Jilin, China
A283	FENGGWS300	II	Jilin,Jilin, China	A326	FENGGWS340	III	Dandong,Liaoning, China

*The accessions are collected from five accumulated temperature zones: Heilongjiang province (I), Jilin province (II), Liaoning province (III), Neimenggu province (IV), North China region (V) & Xinan region (VI). In each zone the temperature remains constant.

Table S3. Significant SNP markers associated with related traits*

No.	Trait	Significant SNPs	Chr	Pos	P Value	$-\log_{10}(P)$	Group	Model
1	SFW	Chr08_29324360	8	29324360	2.29E-07	6.640766	CK	MLMM, super
2	SFW	Chr04_51929177	4	51929177	2.2452E-08	7.648745	AT/CK	BLINK, FarmCPU, MLMM, super, GLM, MLM, CMLM
3	SFW	Chr04_51934424	4	51934424	1.1781E-07	6.928811	AT/CK	MLMM, super, BLINK, FarmCPU
4	SFW	Chr04_52131534	4	52131534	1.4328E-07	6.843804	AT/CK	MLMM, super, BLINK, FarmCPU
5	SFW	Chr04_52367033	4	52367033	1.1385E-07	6.943677	AT/CK	MLMM, super, BLINK, FarmCPU
6	SDW	Chr02_4704111	2	4704111	4.56E-09	8.340861	AT	BLINK, GLM
7	SDW	Chr14_7267405	14	7267405	2.15E-07	6.66829	AT	GLM
8	SDW	Chr14_7324498	14	7324498	3.07E-07	6.513436	AT	GLM
9	SDW	Chr14_7325178	14	7325178	3.02E-07	6.519871	AT	GLM
10	SDW	Chr14_7327054	14	7327054	1.81E-07	6.7432	AT	GLM
11	SDW	Chr14_7333777	14	7333777	2.96E-07	6.528176	AT	GLM
12	SDW	Chr14_7339873	14	7339873	2.20E-07	6.6578	AT	GLM
13	SDW	Chr14_7349175	14	7349175	5.78E-08	7.238409	AT	GLM, BLINK
14	SDW	Chr14_7356062	14	7356062	2.29E-07	6.639446	AT	GLM
15	SDW	Chr14_7358245	14	7358245	3.14E-07	6.502806	AT	GLM
16	SDW	Chr14_7360563	14	7360563	2.09E-07	6.680071	AT	GLM
17	SDW	Chr14_7371500	14	7371500	2.24E-07	6.649972	AT	GLM
18	SDW	Chr14_7375090	14	7375090	9.43E-08	7.025661	AT	GLM
19	SDW	Chr14_7375311	14	7375311	1.91E-07	6.720077	AT	GLM
20	SDW	Chr14_7415401	14	7415401	1.55E-07	6.808744	AT	GLM
21	SDW	Chr14_7415825	14	7415825	2.04E-07	6.69001	AT	GLM
22	SDW	Chr15_10563710	15	10563710	1.02E-07	6.991182	AT	BLINK
23	RDW	Chr01_27555224	1	27555224	2.1839E-07	6.660769	AT/CK	BLINK, FarmCPU
24	RFW	Chr04_34293473	4	34293473	9.512E-08	7.02173	CK	MLMM, super, GLM, MLM
25	RFW	Chr01_38897254	1	38897254	2.3509E-07	6.628773	AT/CK	MLMM, super, CMLM
26	CC	Chr04_8199280	4	8199280	1.834E-07	6.736599	CK	GLM, BLINK
27	CC	Chr04_8201644	4	8201644	2.537E-07	6.595595	CK	GLM
28	CC	Chr16_13644696	16	13644696	1.969E-07	6.705853	CK	BLINK, FarmCPU，MLMM, super, GLM, MLM, CMLM
29	CC	Chr20_25660093	20	25660093	8.432E-08	7.07407	AT, AT/CK	MLMM, super, GLM, MLM, CMLM
30	RN	Chr01_42520188	1	42520188	9.142E-08	7.038938	CK	BLINK, FarmCPU, MLMM, super, GLM, MLM
31	RTN	Chr01_40473337	1	40473337	2.15E-07	6.667484	AT	GLM
32	RTN	Chr01_41287221	1	41287221	2.40E-07	6.620192	AT	GLM
33	RTN	Chr03_3442272	3	3442272	1.12E-07	6.952593	AT	GLM
34	RTN	Chr18_27591088	18	27591088	2.1329E-07	6.671026	AT/CK	BLINK, FarmCPU, MLMM, super
35	RL	Chr01_42520188	1	42520188	9.005E-08	7.045538	CK	BLINK, FarmCPU, MLMM, super, GLM, MLM

*Chr: chromosome; Pos: position; RL: root length; RN: root number; RDW: root dry weight; RFW: root fresh weight; RTN: root tips number; SDW: seedling dry weight; SFW: seedling fresh weight; CC: relative chlorophyll content; CK: control; AT: alkaline stress treatment; AT/CK: the ratio value of the trait under AT in compared to CK.

Table S4. The position of gene underlying the QTLs

No.	QTL	Annotated Gene	Start	Stop
1	*qAT1*	*Glyma.01g113400*	38834287	38837846
2		*Glyma.01g113500*	38934117	38934486
3		*Glyma.01g113600*	38951410	38952227
4		*Glyma.01g113700*	38959028	38959459
5	*qAT4*	*Glyma.04g251900*	51872753	51874821
6		*Glyma.04g252000*	51882953	51887424
7		*Glyma.04g252100*	51892841	51893763
8		*Glyma.04g252200*	51904452	51904610
9		*Glyma.04g252300*	51906741	51909803
10		*Glyma.04g252400*	51915786	51918902
11		*Glyma.04g252500*	51920445	51924637
12		*Glyma.04g252600*	51926107	51928268
13		*Glyma.04g252700*	51934937	51939413
14		*Glyma.04g252800*	51939778	51946490
15		*Glyma.04g252900*	51946270	51951849
16		*Glyma.04g253000*	51964209	51970378
17		*Glyma.04g253100*	51988819	51993371
18		*Glyma.04g253200*	51992841	51993199
19	*qAT14*	*Glyma.14g083600*	7282790	7290881
20		*Glyma.14g083700*	7316262	7318345
21		*Glyma.14g083800*	7326968	7328642
22		*Glyma.14g083900*	7370009	7373267
23		*Glyma.14g084000*	7371098	7371804
24		*Glyma.14g084100*	7372214	7372668
25		*Glyma.14g084200*	7375402	7379417
26		*Glyma.14g084300*	7388630	7392398
27		*Glyma.14g084400*	7400306	7405768
28		*Glyma.14g084500*	7409216	7414343
29		*Glyma.14g084600*	7425379	7427280
30	*qAT18*	*Glyma.18g150300*	27618064	27620103
31	*qAT20*	*Glyma.20g072100*	25593611	25597267
32		*Glyma.20g072200*	25600259	25600778
33		*Glyma.20g072300*	25602439	25603655
34		*Glyma.20g072400*	25658620	25663533
35		*Glyma.20g072500*	25665985	25669495
36		*Glyma.20g072600*	25674460	25692041
37		*Glyma.20g072700*	25692835	25707719
38		*Glyma.20g072800*	25694342	25696996
39		*Glyma.20g072900*	25725323	25727501
